# Mr. Jonathan Hutchinson, F.R.S., on Doctors' Defences

**Published:** 1893-06-24

**Authors:** 


### The Hospital

An Institutional Journal of

Science, Medicine, Nursing, and Philanthropy.

*For Hospitals, Asylums, Infirmaries, Dispensaries, Medical Practitioners, Students, Nurses, and the Charitable Public.*

Vol. XIV.—No. 352.] June 24, 1893.

### Mr. Jonathan Hutchinson, F.R.S., on Doctors' Defences.

There is a great deal of nonsense talked by selfish people about unselfishness. The applauder of self-denial very often means the self-denial of other people for his particular benefit. The man who really desires to live up to the highest level possible for humanity has constantly to face this question, Is there any necessary antagonism between self-preservation and a due regard for the rights of others? The answer to the question is broadly, "No!" If there were, life would not be possible for the thirteen hundred millions of men and women who together inhabit this planet.

There is a consideration which many good people fail to deal logically with, namely, that the man who has no regard for self-preservation leaves the preservation of himself to other people; and humanity is so constituted that other people almost invariably take upon themselves the duty of preserving him the moment he ceases to endeavour to preserve himself. We need not pause to give examples, but the spendthrifts of good families and the idlers among the poorer classes will at once occur to everybody. These have no care for their own preservation; but their families or their parishes burden themselves to provide for their wants with scrupulous carefulness. Self-preservation, then, in its broad sense, is for every individual man and woman one of the most elementary as it is one of the highest and most unselfish of duties.

Mr. Jonathan Hutchinson, F.R.S., presided last Thursday evening at the inaugural dinner of the London and Counties' Medical Protection Society. Mr. Hutchinson is a man of grave and philosophic mind. He felt that the words "Medical Protection" might have a peculiar sound to outsiders, and even to unreflecting medical men. He no doubt remembers and recalls the time when "Protection"—the protection of the agricultural interest—meant, not "protection," but "starvation" to large masses of the industrial poor. Mr. Hutchinson was careful to tell his audience that medical protection has no significance of that character. It is not the protection of the pocket; not an attempt on the part of medical men to enrich themselves at the expense of the sufferings of the general population. It is the defence of reputation, the protection of honour and fair fame which the doctors have in view. This it is which is exposed to such manifold risks in the active practice of medicine; and this which is not only life, but dearer than life, the medical profession is bound to defend at all costs.

It is hardly necessary to remind experienced medical men of the daily risks they run in the pursuit of their avocation. There is scarcely a medical man of twenty years standing anywhere who has not at some time or other felt his "heart stand still" as some possibility of undeserved disgrace and ruin has suddenly yawned like a black earthquake at his feet. Younger physicians and surgeons may be as yet unconscious of their dangers, and may smile at what they consider the timid apprehensiveness of their seniors. But, as Mr. Hutchinson reminded his *confrères*, not one of us, from the most illustrious to the least known, from the greyest senior to the callowest fledgling of the schools, is exempt from these risks and dangers. Unscrupulousness may assail the physician of the most exalted reputation, as it assailed Dr. Broadbent last year. It may also set its fangs in the flesh of the youngest, and destroy him at the very outset of his career.

In union not only is there strength and safety, but there is courage. One man with his back against a wall may make a splendid fight against many enemies. But the least sign of wavering, the smallest mistake, may be the signal for his instant destruction. The same man with a score of valiant and trusted friends by his side will attack and rout a whole regiment of cowardly defamers. The medical practitioner is peculiarly in need of that union with his fellows which gives courage and strength. No other class of men, with perhaps the solitary exception of the Roman Catholic priesthood, are brought into such close and peculiar relations with persons of both sexes as doctors. They may be said to be constantly treading on thin ice. Considering the number of medical men in this country, it is astonishing how few errors of an ethical kind are fallen into, how few occasions the public has for complaint against the medical profession. But though medical men themselves are, as a class, so careful and honourable, their work necessarily lies among all sorts and conditions of men and women; and there are those among all classes who find their interest and their cruellest satisfaction in defaming the innocent and ruining those whose defences they think to be feeble. In such associations as the Medical Protection Society, the timidest and the weakest may find defenders strong enough, experienced enough, and wealthy enough to defend them against every conceivable danger into which honourable men may fall.

Mr. Hutchinson claimed the gratitude of the general public on behalf of the Medical Protection Society. He spoke with knowledge. We have admitted that the Society defends doctors in the first instance; and we have shown that in so doing it is both exercising a right, and practising an elementary duty, the prime duty of the preservation of their honour. But the Association really defends the public as much, and almost more than it defends its own constituents. The most cynical student of human nature will admit that knaves are injurious to the body politic! Is a medical knave any less injurious than any other knave? A medical association which strives to put an end to ignorant and dishonest medical practice is doing the public a great service, and is entitled to corresponding gratitude. We have no sympathy whatever with those timid practitioners who think that the public will not give us credit for disinterestedness, and who therefore refrain from the useful and necessary work of exposing knaves. For our part, we do not care whether the public gives us credit or not. The medical interests, the health interests of the population belong to us by the right of special capacity to deal with them, as much as the religious interests of the people naturally belong to the Universal Church. We should be less than Englishmen, we should be cowards and poltroons if, because of the irresponsible cynicism of the uninformed, we should withhold our hands from just and necessary public duty. Every sensible man and woman, every man and woman who judges other people from the standpoint of personal integrity and rightmindedness, will not only admit our claim to be defenders of the health and the medical interests of the public, but would declare us to be recreant to our special knowledge and to our British manhood if we did not defend those interests. The London and Counties Medical Protection Society, under the presidency of Mr. Jonathan Hutchinson, boldly raises its flag against quackery and knavery of all kinds, and proclaims war to the knife.

As we are writing chiefly for our professional brethren we may be permitted to describe in a word the present position of the Society. To begin with, it is less than a year old; it already numbers upwards of 700 members, many of whom, like its president, Mr. Jonathan Hutchinson, are men of the highest distinction; and last, but not least, it has paid its way, and has a little balance to its credit at the close of its first year. An organisation which in its earliest seedtime can thus show a balance in its favour has the fairest possible promise of success and abundance when the period of reaping arrives. The honorary officers of the Society, Dr. George Heron, Dr. Mead, and Dr. Hugh Woods have piloted it successfully through its first year, and have earned the gratitude of the whole profession by their strenuous and disinterested labours. We have sincere satisfaction in presenting the merits of this new Association to the favourable consideration of our readers.

